# Intravenous Fat Emulsion Administration and Short-Term Lipid Profiles Under a Standardized Clinical Pathway After Gastrectomy: A Retrospective Observational Study

**DOI:** 10.3390/nu18213566

**Published:** 2026-10-28

**Authors:** Kazuo Kobayashi, Satoshi Ida, Masanari Tsuji, Takeshi Aoyama, Takashi Yokokawa, Yoshikazu Tateai, Shuichi Watabe, Hisanori Shimizu, Souya Nunobe, Masakazu Yamaguchi

**Affiliations:** 1 Department of Pharmacy, Cancer Institute Hospital, Japanese Foundation for Cancer Research, 3-8-31 Ariake, Koto-ku, Tokyo 135-8550, Japan; takeshi.aoyama@jfcr.or.jp (T.A.); yoshikazu.tateai@jfcr.or.jp (Y.T.); shuichi.watabe@jfcr.or.jp (S.W.); hisanori.shimizu@jfcr.or.jp (H.S.); masakazu.yamaguchi@jfcr.or.jp (M.Y.); 2 Department of Gastroenterological Surgery, Cancer Institute Hospital, Japanese Foundation for Cancer Research, Koto-ku, Tokyo 135-8550, Japan; s-ida@kumamoto-u.ac.jp (S.I.); souya.nunobe@jfcr.or.jp (S.N.); 3 UNISMILE Pharmacy Komagome Hospital Front Store, UNISMILE Co., Ltd., 5-50-1 Sendagi, Bunkyo-ku, Tokyo 113-0022, Japan; m.tsuji.142857@gmail.com; 4 Department of Clinical Pharmacology, School of Pharmacy, Tokyo University of Pharmacy and Life Sciences, Hachioji, Tokyo 192-0392, Japan; tyokoka@toyaku.ac.jp

**Keywords:** fat emulsion, gastrectomy, lipid abnormalities, triglycerides, postoperative nutritional management

## Abstract

Background/Objectives: Real-world data on intravenous fat emulsion administration and postoperative lipid profiles under standardized clinical pathways are limited. We described administration practices and short-term lipid profiles after gastrectomy. Methods: This single-center retrospective study included patients who underwent gastrectomy for gastric cancer between August and December 2020 and received postoperative fat emulsion. The pathway specified 50 g/day of a 20% soybean oil-based emulsion, starting on postoperative day (POD) 1, generally for five days. Dose, infusion duration, weight-adjusted infusion rate, and serum triglyceride (TG) and total cholesterol (T-Cho) levels were assessed descriptively. Lipid elevations were graded using the Common Terminology Criteria for Adverse Events version 5.0. Results: Among 89 patients, 85 received fat emulsion and were analyzed. The median infusion rate was 0.16 g/kg/h (range, 0.09–0.34); 83 patients (97.6%) received rates >0.1 g/kg/h, including 67 (78.8%) at >0.1 to ≤0.2 g/kg/h. No grade ≥2 TG or T-Cho elevations were observed in available measurements on POD 3 or POD 7. Median TG was 107.5 mg/dL before surgery and 119 mg/dL on POD 7; corresponding T-Cho values were 188 and 146 mg/dL. Outpatient TG measurements were available for only four patients. Conclusions: These findings describe short-term lipid profiles under a fixed-dose postoperative nutritional pathway. They do not establish the safety of infusion rates exceeding 0.1 g/kg/h or define a safe upper infusion-rate threshold.

## 1. Introduction

Gastric cancer remains a major global health burden [1], and gastrectomy is a standard curative treatment for resectable disease [2]. Appropriate postoperative nutritional management supports recovery. Enhanced recovery after surgery (ERAS) protocols incorporate early nutritional support [3]. Intravenous fat emulsions provide a concentrated energy source and essential fatty acids when oral or enteral intake is insufficient [4].

Hypertriglyceridemia is a concern during intravenous fat emulsion administration, particularly when lipid clearance is impaired. Surgical and metabolic stress may alter lipid utilization and clearance [5,6]. The Japanese Society for Parenteral and Enteral Nutrition (JSPEN) guidelines recommend an infusion rate of ≤0.1 g/kg/h [7]. Broader parenteral nutrition guidance emphasizes attention to lipid dose and metabolic tolerance [8,9].

In routine postoperative care, institutional clinical pathways may specify fixed lipid doses and infusion durations. When these are not adjusted for body weight, weight-adjusted infusion rates can vary substantially. Detailed data on administration practices and accompanying lipid profiles under such pathways remain limited.

Therefore, this retrospective observational study aimed to describe real-world intravenous fat emulsion administration practices and short-term postoperative lipid profiles in patients undergoing gastrectomy under a standardized institutional clinical pathway.

## 2. Materials and Methods

### 2.1. Study Design and Eligible Patients

This retrospective observational study was conducted at the Cancer Institute Hospital Ariake to describe intravenous fat emulsion administration practices and postoperative lipid profiles in patients undergoing gastrectomy for gastric cancer. The perioperative management protocol was consistent with that used in our previously reported study on postoperative liver dysfunction and was based on a standardized institutional clinical pathway routinely applied at our hospital [10].

Patients who underwent radical gastrectomy for gastric cancer between 1 August 2020 and 31 December 2020 were included in this study. All patients received early postoperative nutritional support according to the institutional pathway. Postoperative parenteral nutrition included administration of an amino acid-containing infusion solution together with a 20% soybean oil-based lipid emulsion (Intralipos^®^ 20%, 250 mL; Otsuka Pharmaceutical Factory, Inc., Naruto, Japan), corresponding to 50 g of lipid per day. According to the institutional clinical pathway, lipid emulsion administration was scheduled to begin on postoperative day (POD) 1 at 50 g/day for five consecutive days in principle; however, the duration of administration could be shortened or extended according to the patient’s postoperative clinical condition. Consequently, the total lipid dose could vary among patients. In routine clinical practice, the infusion rate of the lipid emulsion was not adjusted according to body weight.

The standardized clinical pathway, previously described in detail [10], targeted approximately 1500 kcal/day and included BFLUID^®^ (Otsuka Pharmaceutical Factory, Inc., Naruto, Japan; 2000 mL/day; approximately 60 g amino acids with glucose, electrolytes, and vitamin B1) and Intralipos^®^ 20% (250 mL/day, 50 g lipids), generally infused over approximately five hours. These amounts describe the planned pathway, not measured individual nutrient intake.

Oral intake was initiated and advanced in parallel with parenteral nutrition. Early oral intake was encouraged according to the clinical pathway; however, the timing and progression of diet advancement were determined by the attending surgeons based on each patient’s postoperative condition.

The institutional protocol initiated oral fluids on POD 1 and advanced to a semi-liquid diet by approximately POD 3, followed by gradual advancement as tolerated. An elemental supplement (Elental^®^; EA Pharma Co., Ltd., Tokyo, Japan; 250 kcal twice daily) was scheduled from POD 3 [10]. Actual calorie intake and adherence to the nutritional plan were not assessed in this dataset.

### 2.2. Data Collection

Clinical and laboratory data were retrospectively collected from electronic medical records. Blood samples were routinely obtained on the day immediately before surgery, on postoperative day 3 and POD 7 (±1 day), and at the first outpatient visit after hospital discharge, in accordance with routine clinical practice at our institution. During hospitalization, samples were generally obtained in the morning before breakfast; however, at the first outpatient visit, samples were not necessarily collected under fasting conditions, and fasting status was not systematically recorded.

During hospitalization, samples were usually drawn in the early morning, at approximately 06:00 or 08:00 as part of routine care. Individual infusion-stop and blood-draw timestamps were not available for this analysis. Since lipid administration was generally scheduled for POD 1–5, POD 3 sampling usually occurred during the treatment period, whereas POD 7 predominantly represents assessment after planned treatment completion.

Laboratory tests were performed at all scheduled time points unless unavailable due to early discharge or other clinical circumstances. Missing laboratory data were excluded from the respective analyses and were not imputed.

Serum lipid parameters, including triglyceride (TG) and total cholesterol (T-Cho) levels, were assessed at each time point and used to evaluate postoperative lipid abnormalities. In addition, information regarding intravenous fat emulsion administration, including dose and infusion duration, was extracted to calculate the actual infusion rate for each patient. Demographic data and perioperative clinical variables were also collected for analysis.

### 2.3. Endpoints

The endpoints were to characterize real-world intravenous fat emulsion administration after gastrectomy, including actual infusion rates and total administered doses, and to describe short-term postoperative changes in serum TG and T-Cho levels.

Postoperative lipid abnormalities were assessed using serum TG and T-Cho levels according to the Common Terminology Criteria for Adverse Events (CTCAE) version 5.0. Elevations in TG or T-Cho corresponding to CTCAE grade 1 or higher were defined as lipid abnormalities. Changes in TG and T-Cho levels over time were descriptively evaluated.

The weight-adjusted infusion rate (g/kg/h) was calculated from the administered lipid dose (g), infusion duration (h), and patient body weight (kg). Infusion rates were evaluated relative to a benchmark of 0.1 g/kg/h, based on the JSPEN guidelines, which recommend an infusion rate of ≤0.1 g/kg/h [7].

### 2.4. Statistical Analysis

This study was conducted as a descriptive analysis to characterize real-world administration practices of intravenous fat emulsions and short-term postoperative lipid profiles in patients undergoing gastrectomy for gastric cancer.

Continuous variables were summarized as medians and ranges. Categorical variables were summarized as counts and percentages. Lipid concentrations and grade distributions were summarized using the available observations at each time point. Laboratory test availability differed by analyte and time point. The POD 3 TG analysis included 75 evaluable patients. The reasons for unavailable POD 3 total cholesterol values and outpatient TG measurements could not be reliably determined from the retrospective dataset. Missing values were not imputed. An exploratory descriptive analysis compared serum TG profiles between patients with lipid infusion rates >0.2 g/kg/h (*n* = 16) and ≤0.2 g/kg/h (*n* = 69). TG concentrations at baseline and POD 7 (±1 day), as well as within-patient changes among those with paired measurements, were summarized using medians. No inferential statistical analyses were performed.

## 3. Results

### 3.1. Patient Enrollment

**Figure 1:**
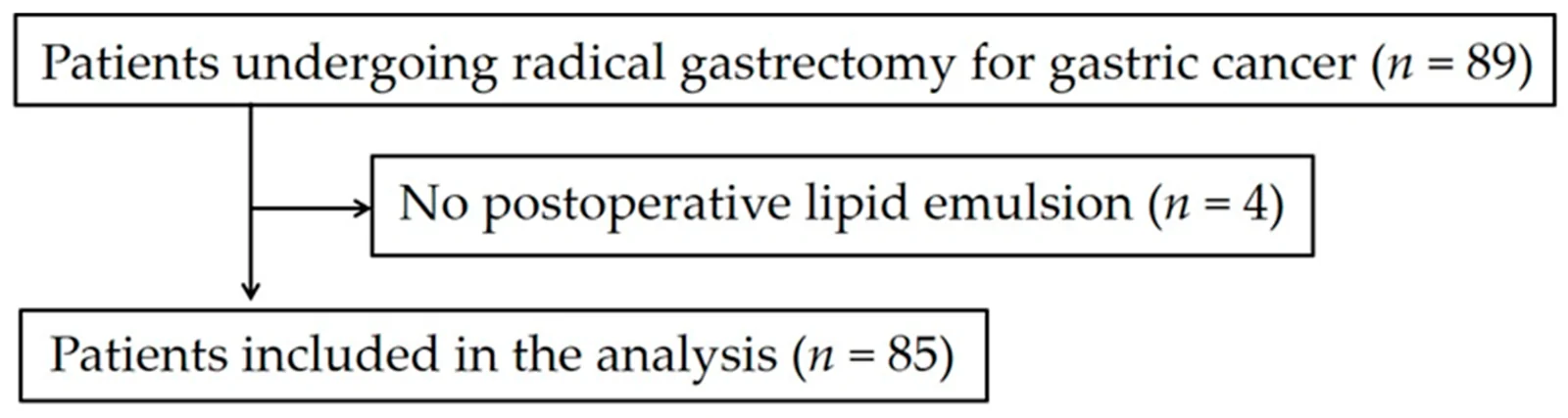
Patient selection. Four patients who did not receive postoperative lipid emulsion were excluded.

During the study period, 89 patients underwent radical gastrectomy for gastric cancer. Among them, 4 patients who did not receive postoperative lipid emulsion administration were excluded. Consequently, 85 patients were eligible for the final analysis (Figure 1). The baseline characteristics of the eligible patients are shown in Table 1.

Among the 85 patients, surgical access was open in 28 (32.9%), laparoscopic in 56 (65.9%), and thoracoabdominal in one (1.2%); 22 of the laparoscopic procedures were robot-assisted. The resection types were distal gastrectomy (*n* = 50), total gastrectomy (*n* = 23, including four completion gastrectomies and one with lower esophageal resection), pylorus-preserving gastrectomy (*n* = 5), proximal gastrectomy (*n* = 5), and partial gastrectomy (*n* = 2). Reconstruction methods are summarized in Table 1. Clinical stage refers to the gastric cancer stage.

### 3.2. Dose and Infusion Rate of Fat Emulsion

The total lipid dose administered up to POD 7 was 250 g in 68 patients (80.0%), while the remaining patients received lower or higher cumulative doses (Table 2). The median infusion rate of intravenous fat emulsion was 0.16 g/kg/h (range, 0.09–0.34 g/kg/h). Fat emulsion was administered at a rate >0.1 g/kg/h in 83 patients (97.6%), whereas rates >0.3 g/kg/h were observed in two patients (2.4%). The infusion duration per bottle was largely consistent across patients at approximately 5 h.

### 3.3. Postoperative Lipid Profiles

**Figure 2:**
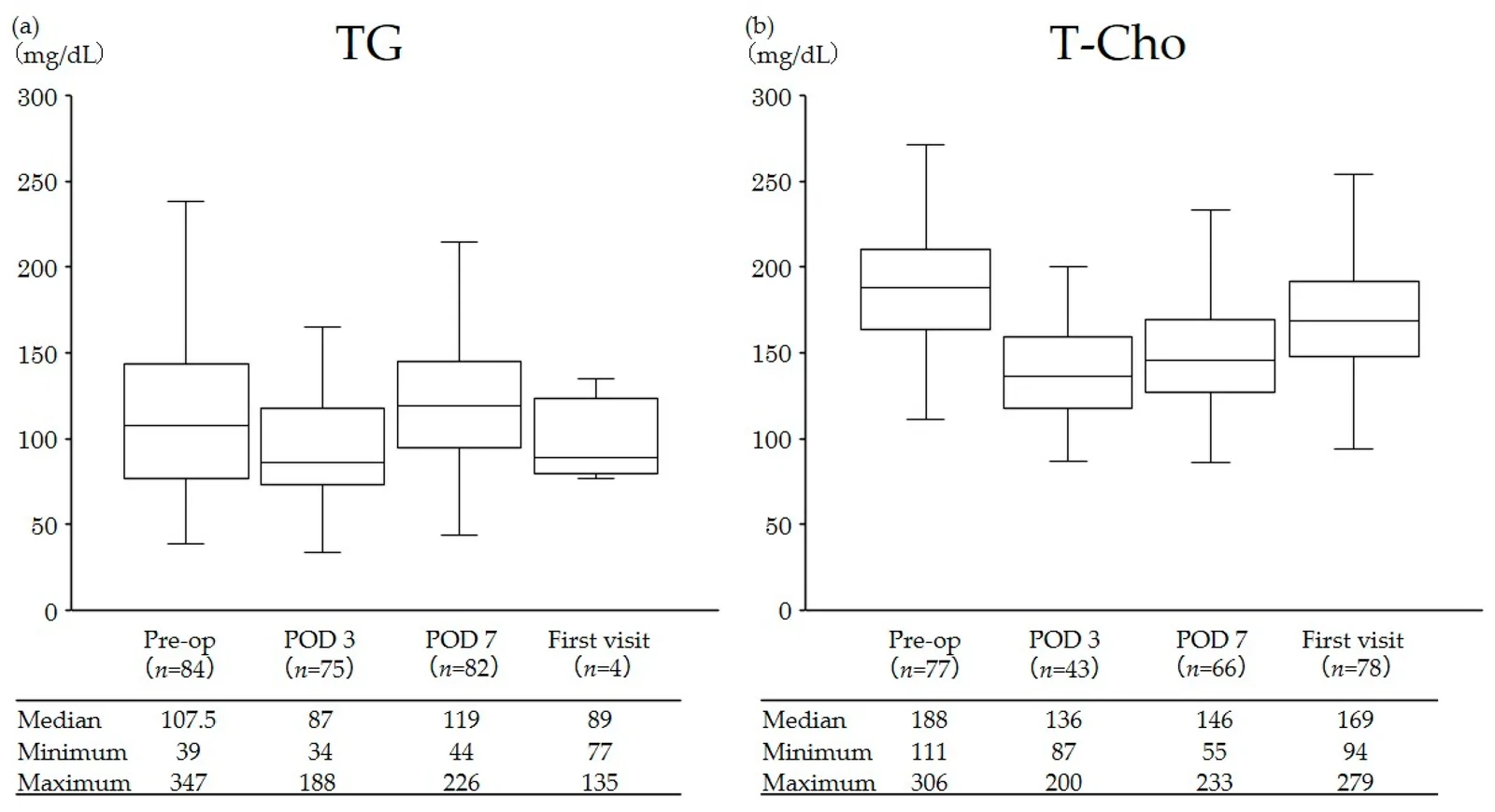
Serum lipid concentrations before and after surgery: (**a**) triglycerides (TG); (**b**) total cholesterol (T-Cho). Boxes indicate the interquartile range, and horizontal lines indicate medians. Whiskers extend to the minimum and maximum values excluding outliers. Sample sizes and descriptive statistics are shown for each time point. Pre-op, preoperative; POD, postoperative day; first visit, first outpatient visit after discharge.

Changes in serum TG and T-Cho levels are shown in Figure 2. Serum TG levels showed a modest increase during the postoperative period, particularly on POD 7, whereas T-Cho levels decreased after surgery and gradually recovered thereafter. Median TG levels increased from 107.5 mg/dL preoperatively to 119 mg/dL on POD 7, whereas median T-Cho levels decreased from 188 mg/dL preoperatively to 146 mg/dL.

No CTCAE grade ≥2 TG or T-Cho elevations were observed in available measurements on POD 3 or POD 7. Grade 1 TG elevation was recorded in 14/84 patients (16.7%) preoperatively, 5/75 (6.7%) on POD 3, and 20/82 (24.4%) on POD 7 (Table 3). Corresponding grade distributions for T-Cho are shown in Table 4. Outpatient TG measurements were available in only four patients. An exploratory analysis stratified 16 patients with infusion rates >0.2 g/kg/h and 69 with rates ≤0.2 g/kg/h. The respective preoperative TG medians were 106 (*n* = 15) and 108 mg/dL (*n* = 69), while POD 7 (±1 day) medians were 125 (*n* = 15) and 117 mg/dL (*n* = 67). Median within-patient changes from baseline to POD 7 (±1 day) were +17 mg/dL (*n* = 15) and +8 mg/dL (*n* = 67), respectively (Table 5). These descriptive differences should not be interpreted as a causal effect of infusion rate.

## 4. Discussion

This study described real-world intravenous fat emulsion administration and short-term postoperative lipid profiles in patients undergoing gastrectomy under a standardized institutional clinical pathway. Most patients received weight-adjusted infusion rates between 0.1 and 0.2 g/kg/h, whereas rates >0.3 g/kg/h were uncommon. During the early postoperative period, no CTCAE grade ≥2 elevations in TG or T-Cho were observed on POD 3 or POD 7. These findings provide descriptive real-world data on lipid profiles accompanying short-term fat emulsion administration after gastrectomy.

Serum TG levels showed a modest increase by POD 7, whereas T-Cho levels decreased after surgery. These changes may reflect transient alterations in lipid metabolism associated with surgical stress, postoperative insulin resistance, and perioperative nutritional conditions [5,6]. However, these changes should be interpreted as a description of postoperative lipid profiles under the institutional clinical pathway rather than as evidence of the metabolic safety of the regimen.

The proportion of grade 1 TG elevations increased from 5/75 (6.7%) on POD 3 to 20/82 (24.4%) on POD 7. Since the denominators differed, this comparison does not demonstrate progression within the same patients. Moreover, POD 7 largely followed completion of the planned lipid infusion and cannot capture transient changes during treatment.

The 0.1 g/kg/h benchmark used in this study was based on the JSPEN guidelines [7]. ASPEN recommendations specify a maximum infusion rate of 0.11 g/kg/h for soybean oil-based lipid emulsions [8]. In this cohort, 83/85 patients (97.6%) received infusion rates above the JSPEN benchmark, although most received rates >0.1 to ≤0.2 g/kg/h and only two patients received rates >0.3 g/kg/h. These findings describe actual administration practices and short-term lipid profiles under the institutional pathway and should not be interpreted as establishing the safety of infusion rates above the recommended benchmark.

Fixed-dose clinical pathways can simplify postoperative nutritional delivery but produce different weight-adjusted infusion rates across patients. Our findings quantify this variation and describe the accompanying short-term lipid profiles. Together with our previous report on postoperative liver dysfunction under the same institutional pathway [10], they provide complementary information for reviewing local nutritional practice. They do not define an optimal or safe upper infusion-rate threshold.

The previous liver-dysfunction study [10] included 170 patients operated on during January–July 2020, whereas this study enrolled patients operated on during August–December 2020; consequently, there is no cohort overlap by enrollment period. In the previous cohort, a lipid infusion rate >0.1 g/kg/h was not significantly associated with POD 7 AST elevation (adjusted odds ratio 0.87, 95% confidence interval 0.30–2.45; *p* = 0.793). These findings relate to a distinct outcome and population. Beyond fixed-dose pathways, structured nursing assessment and monitoring complexity may inform individualized care; the use of standardized nursing datasets for characterizing care complexity has been proposed in methodological work [11], without direct evidence on lipid emulsion safety.

This study has several limitations. First, it was a retrospective, single-center observational study conducted under a specific institutional clinical pathway, which may limit the generalizability of the findings. Second, no untreated or prospectively assigned infusion-rate comparator group was available; therefore, causal relationships between fat emulsion administration, infusion rate, and postoperative lipid changes cannot be determined. Third, only two patients received infusion rates >0.3 g/kg/h, precluding evaluation of markedly higher infusion rates or determination of an upper safe infusion-rate threshold. Fourth, lipid profiles were evaluated primarily during the early postoperative period, and TG measurements at the first outpatient visit were available in only a small number of patients. The timing of the first outpatient visit varied among patients and could not be reliably quantified from the available dataset. Therefore, post-discharge lipid trends, including delayed or persistent hypertriglyceridemia, could not be adequately assessed.

The small exploratory high-rate subgroup and the lack of adjustment for operative approach, nutritional status, inflammation, and concomitant intake preclude causal interpretation. Surgical approaches, resection types, and reconstruction methods were available from the source dataset and are summarized in Table 1. Detailed preoperative liver and pancreatic laboratory profiles, validated nutritional screening scores, and postoperative ICU admission data were not systematically collected for this analysis. In addition, nonfasting outpatient sampling may produce higher TG values than fasting sampling, while only four outpatient TG results were available, limiting post-discharge interpretation.

In addition, the number of available measurements differed across time points. Comparisons of medians therefore do not necessarily represent within-patient changes. Fasting status at the first outpatient visit was not systematically recorded, further limiting interpretation of outpatient lipid values. The absence of grade ≥2 elevations at scheduled measurements does not exclude transient elevations between sampling times or other adverse effects of lipid administration.

## 5. Conclusions

This retrospective study provides real-world data on intravenous fat emulsion administration and short-term postoperative lipid profiles under a standardized clinical pathway after gastrectomy. These findings may help inform the evaluation of standardized postoperative nutritional pathways but do not establish the safety of infusion rates exceeding current guideline recommendations.

## Figures and Tables

**Table 1:** Patient characteristics.

Characteristic	N = 85
Sex, male/female	63/22
Age, years	68 (36–89)
Preoperative body weight, kg	60.8 (38–87)
Preoperative body mass index, kg/m^2^	22.6 (16–29)
History of lipid-lowering medication use, *n* (%)	22 (25.9)
Clinical stage of gastric cancer I/II/III/IV	49/18/15/3
Surgical access: open/laparoscopic/thoracoabdominal, n	28/56/1
Robot-assisted procedures (subset of laparoscopic), *n* (%)	22 (25.9)
Resection: distal/total/pylorus-preserving/proximal/partial, n	50/23/5/5/2
Reconstruction: Roux-en-Y antecolic/retrocolic/Billroth I/Billroth II/esophagogastrostomy/other/not recorded, *n*	44/14/12/1/5/3/6

Values are medians (ranges) or counts unless otherwise indicated. Resection and reconstruction categories each total 85 patients; robot-assisted procedures are included in the laparoscopic category. The total gastrectomy group (*n* = 23) includes four patients who underwent completion gastrectomy and one patient who underwent total gastrectomy with lower esophageal resection.

**Table 2:** Dose and infusion rate of lipid emulsion.

Administration Parameter	Patients, *n* (%)
Infusion rate, g/kg/h	
≤0.1	2 (2.4)
>0.1 to ≤0.2	67 (78.8)
>0.2 to ≤0.3	14 (16.5)
>0.3	2 (2.4)
Cumulative lipid dose through POD 7, g	
100	3 (3.5)
150	1 (1.2)
200	1 (1.2)
250	68 (80.0)
300	0 (0.0)
350	11 (12.9)
500	1 (1.2)

N = 85. Percentages may not sum to 100% because of rounding. POD, postoperative day.

**Table 3:** Distribution of triglyceride elevation grades.

Grade	Pre-op (*n* = 84)	POD 3 (*n* = 75)	POD 7 (*n* = 82)	First Outpatient Visit (*n* = 4)
Grade 0	67 (79.8)	70 (93.3)	62 (75.6)	4 (100.0)
Grade 1	14 (16.7)	5 (6.7)	20 (24.4)	0 (0.0)
Grade 2	3 (3.6)	0 (0.0)	0 (0.0)	0 (0.0)
Grade 3	0 (0.0)	0 (0.0)	0 (0.0)	0 (0.0)
Grade 4	0 (0.0)	0 (0.0)	0 (0.0)	0 (0.0)

Values are *n* (%), with available measurements at each time point as the denominator. Grading follows CTCAE version 5.0. Grade 0 denotes no graded elevation. Pre-op, preoperative; POD, postoperative day. Percentages may not sum to 100% because of rounding.

**Table 4:** Distribution of total cholesterol elevation grades.

Grade	Pre-op (*n* = 77)	POD 3 (*n* = 43)	POD 7 (*n* = 66)	First Outpatient Visit (*n* = 78)
Grade 0	63 (81.8)	43 (100.0)	65 (98.5)	73 (93.6)
Grade 1	13 (16.9)	0 (0.0)	1 (1.5)	5 (6.4)
Grade 2	1 (1.3)	0 (0.0)	0 (0.0)	0 (0.0)
Grade 3	0 (0.0)	0 (0.0)	0 (0.0)	0 (0.0)
Grade 4	0 (0.0)	0 (0.0)	0 (0.0)	0 (0.0)

**Table 5:** Exploratory triglyceride profiles according to weight-adjusted lipid infusion rate. Descriptive subgroup analysis of available measurements.

TG profile	>0.2 g/kg/h (*n* = 16)	≤0.2 g/kg/h (*n* = 69)
Preoperative, median, mg/dL	106 (*n* = 15)	108 (*n* = 69)
POD 7, median, mg/dL	125 (*n* = 15)	117 (*n* = 67)
Paired change from preoperative to POD 7, median, mg/dL	+17 (*n* = 15)	+8 (*n* = 67)

Values are medians (mg/dL), with the number of available observations shown in parentheses. Paired changes were calculated only for patients with both preoperative and POD 7 (±1 day) values. This analysis is descriptive.

## Data Availability

Data supporting the findings of this study are available upon request from the corresponding author. The data are not publicly available because of privacy and ethical restrictions.

## Abbreviations

CTCAE, Common Terminology Criteria for Adverse Events; ERAS, enhanced recovery after surgery; IRB, institutional review board; POD, postoperative day; T-Cho, total cholesterol; TG, triglycerides.
